# The Amines Treatment of Sewage

**Published:** 1889-09-28

**Authors:** 


### The Amines Treatment of Sewage.

Now that a "policy of sewage" no longer seems a thing to make a mock of, but one vitally important to the nation, it does not seem strange that the Lord Mayor of London should go to Wimbledon to see the sewage of the town treated by a new process. As has been often pointed out in these columns, to render sewage inoffensive and innocuous is only fighting half the battle of man *versus* microbe. Nature will not be satisfied till it is returned to her to be rendered beneficial. Our present method of settling the sewage question is only less bad than not settling it at all. We throw it into the sea or into any river that happens to be near, and leave stream and tide to do with it what they will. We starve the fields and we poison the fishes. This is bad; for though it is better that fish should die than men, we cannot be sure that in refusing to the earth the nutriment it requires to make it fertile, we are not conniving at poverty and want, that lead to death, among our fellow-creatures.

Most of the processes that have been suggested for the treatment of the sewage of our large towns have attempted to fulfil only the first part of our requirements—that of rendering it harmless. It is to the credit of Mr. Wollheim, the inventor of the Amines process, that while making this his primary object, he has not forgot the second desideratum, though he wisely makes no calculation of the possible profit that may accrue from this, in calculating the cost of the treatment.

The Amines process derives its name from the compounds of ammonia used in it. It is the organic bases of these amines which, combined with milk of lime, disinfect the sewage. At Wimbledon, they appear in the homely form of herring brine, which, when mixed with the lime, forms a gaseous, salt-smelling reagent, called amminol. Amminol is a stirring and vigorous disinfectant. When introduced among the sewage it stirs up a brief commotion, which terminates in the complete extirpation of all disease-breeding germs. The solid particles subside, and the fluid that floats on top is clear, and absolutely free from microbes of any sort. The process does not take long. In a settling-tank six feet deep all is over in half an hour. The amminol has done its beneficent work, and in place of offensive and dangerous matter you have a sterile effluent, and a residuum, or sludge, which can be made of practical value.

Dr. Klein, who is less likely than any man in England to miss a microbe, reports that in a sample of sewage, which contained more than two million micro-organisms to the cubic centimetre, there was not one left in the effluent that was produced by the Amines process. Yet nothing has been taken from the sewage in the process. All the ingredients which are of value to vegetable life are still present, but they have fallen to the bottom of the tank. Let this residuum be pressed into cake, or dried into powder, and it forms a good manure, conveniently portable, which it was not before, and undeniably of market value.

One point remains to be considered—the cost of the process. Though herring brine is not an expensive commodity, a process which requires tanks, machinery, and chemicals must cost more than the simple method of sending the sewage into the Thames to defile its stream. Mr. Wollheim calculates the cost at about ½d. to ¾d. for every 1,000 gallons purified. Not a great sum, certainly, but then the sewage of London amounts to a hundred and thirty million gallons daily. This implies a daily expense of about £330, or about £125,000 per annum. This implies increased taxation, but how much? About 7½d. for each person in London. This rate can hardly be regarded as "intolerable and not to be borne." And this cost is calculated on the sheer expense, without deduction of any sort for the profit that would accrue from the sale of the sludge. That the sludge would sell, and that there would be a good demand for it, we may take as certain, and if British farmers would not purchase so good a manure when it was made conveniently attainable, they are less sensible than we take them to be. The cost of the Amines process would thus thus be reduced to a still more moderate sum, even if it be ash to opine that it might be changed to profit. The subject has been brought before Lord Rosebery and the members of the London County Council. These gentlemen will not act rashly, nor without consulting hygienists; but whatever conclusion they come to with regard to this particular process, we hope they will determine on a "policy of sewage" that will transform a long-standing nuisance into a blessing.

